# Nitrogen uptake kinetics and saltmarsh plant responses to global change

**DOI:** 10.1038/s41598-018-23349-8

**Published:** 2018-03-29

**Authors:** Grace M. Cott, Joshua S. Caplan, Thomas J. Mozdzer

**Affiliations:** 1 Smithsonian Environmental Research Center, 647 Contees Wharf Road, Edgewater, Maryland 21037 USA; 2 School of Biological, Earth and Environmental Sciences, University College Cork, Distillery Fields Campus, Cork, Ireland; 3 Department of Biology, Bryn Mawr College, 101 North Merion Avenue, Bryn Mawr, Pennsylvania 19010 USA; 4 Present Address: Department of Landscape Architecture & Horticulture, Temple University, 580 Meetinghouse Road, Ambler, Pennsylvania 19002 USA

## Abstract

Coastal wetlands are important carbon sinks globally, but their ability to store carbon hinges on their nitrogen (N) supply and N uptake dynamics of dominant plant species. In terrestrial ecosystems, uptake of nitrate (NO_3_^−^) and ammonium (NH_4_^+^) through roots can strongly influence N acquisition rates and their responses to environmental factors such as rising atmospheric CO_2_ and eutrophication. We examined the ^15^N uptake kinetics of three dominant plant species in North American coastal wetlands (*Spartina patens*, C_4_ grass; *Phragmites australis*, C_3_ grass; *Schoenoplectus americanus*, C_3_ sedge) under ambient and elevated CO_2_ conditions. We further related our results to the productivity response of these species in two long-term field experiments. *S. patens* had the greatest uptake rates for NO_3_^−^ and NH_4_^+^ under ambient conditions, suggesting that N uptake kinetics may underlie its strong productivity response to N in the field. Elevated CO_2_ increased NH_4_^+^ and NO_3_^−^ uptake rates for *S. patens*, but had negative effects on NO_3_^−^ uptake rates in *P. australis* and no effects on *S. americanus*. We suggest that N uptake kinetics may explain differences in plant community composition in coastal wetlands and that CO_2_-induced shifts, in combination with N proliferation, could alter ecosystem-scale productivity patterns of saltmarshes globally.

## Introduction

Anthropogenic activities enrich the atmosphere with CO_2_, but the extent to which the biosphere can absorb this increase remains uncertain^1,2^. Saltmarshes play a disproportionally large role in global carbon storage, sequestering up to 87 Tg of carbon per year worldwide despite comprising less than 0.5% of the Earth’s land area^3^. However, the capacity of a given coastal wetland to store carbon hinges primarily on its nitrogen (N) supply and the N uptake dynamics of its dominant plant species^4–6^. In terrestrial ecosystems including coastal wetlands, root absorption of nitrate (NO_3_^−^) and ammonium (NH_4_^+^) strongly influences the rate of N acquisition by plants and how this varies in response to environmental factors^7–9^. Nitrogen availability also constrains ecosystem productivity, with N enrichment leading to species shifts that can favor both native^10^ and introduced species^11,12^. However, saltmarsh species differ in their N metabolizing activities, with rates influenced by the chemical form of N and its concentration in the substrate^13–15^, oxygenation of the rhizosphere^16^, salinity conditions^17^, and the presence of toxins such as sulfide^18^. Understanding the kinetics of root nitrogen uptake and potential differences among foundation species is therefore crucial for predicting saltmarsh ecosystems’ potential for carbon storage as atmospheric CO_2_ levels rise.

While the effects of elevated CO_2_ on plant productivity are well studied in a variety of ecosystems^19,20^ including saltmarshes^21,22^, our understanding of these responses is primarily based on changes in aboveground biomass, CO_2_ assimilation^23,24^, and shifts in species composition that reflect competition between plant functional groups^10^. Little attention has been paid to belowground mechanisms such as those associated with N acquisition or how they could be altered by global change. In an elevated CO_2_ environment, plants generally experience a decline in tissue N concentration^25^, due to either a dilution of Rubisco^26–28^ or a reduction of transpiration-driven mass flow of N through soils^29^; declines have been measured despite ample N supply^30,31^. Recent evidence also suggests that, at least for C_3_ plants under NO_3_^−^ nutrition, CO_2_ enrichment can decrease tissue N directly by slowing growth and inhibiting shoot NO_3_^−^ assimilation^32^. However, the direct effects of elevated CO_2_ on N uptake have only been investigated in a small number of studies, making it difficult to generalize how different species or functional groups may adjust N uptake in response to elevated CO_2_.

Although photosynthetic pathways typically determine plant physiological responses to elevated CO_2_, the circumstances under which these physiological differences translate into a change in N uptake kinetics is not yet clear. While N acquisition is generally not affected by elevated CO_2_ in C_4_ plants, C_3_ plants show variable patterns in uptake parameters under elevated CO_2_ (e.g., V_max_ or K_m_)^9^. For example, in studies of temperate forest trees, the effect of elevated CO_2_ on NH_4_^+^ root uptake capacity was species dependent, ranging from +215% in *Acer negundo* to −40% in *Quercus macrocarpa*^33^. In related studies, elevated CO_2_ increased the maximum rate of NO_3_^−^ uptake, specifically in *Pinus ponderosa*, *Bouteloua eriopoda* and *Pinus taeda*^34–37^. Other studies have found no significant effect of elevated CO_2_ on NO_3_^−^ or NH_4_^+^ uptake rates, namely in *Pinus taeda, Prosopis glandulosa, Ceratonia siliqua*, and several herbaceous species^35–40^. Furthermore, effects of elevated CO_2_ are not limited to N uptake rates. In the case of a C_3_ tropical seagrass, *Halodule uninervis*, elevated CO_2_ inhibited NO_3_^−^ assimilation and NO_3_^−^ nutrition alone did not enhance the CO_2_ response^41^. One proposed explanation for the species-specific effects of elevated CO_2_ on NO_3_^−^ assimilation in C_3_ plants is that elevated CO_2_ concentrations decrease photorespiration, thereby decreasing the amount of reductant (NADH) available to support NO_3_^−^ reduction to NO_2_^−^ in the first step of NO_3_^−^ assimilation^42^. In contrast, the C_4_ carbon fixation pathway generates sufficient quantities of reductants in the cytoplasm of mesophyll cells, thus avoiding the inhibitive effect of elevated CO_2_ on NO_3_^−^ assimilation^43^.

The physiological capacity for nitrogen uptake and assimilation may provide a key mechanistic explanation for interspecific differences in sensitivity to CO_2_ and N addition^9^. For example, N uptake dynamics may explain why N addition can favor coastal wetland species that do not respond strongly to elevated CO_2_ (i.e., *Spartina patens*), ultimately negating the enhanced productivity response at the ecosystem-level^10^. In the context of anthropogenically-induced changes to the carbon and nitrogen cycles in wetland ecosystems, information on physiological responses of N uptake to elevated CO_2_ could be highly relevant for understanding these species shifts and how they influence critical ecosystem-level phenomena such as resilience to sea level rise and carbon sequestration^44^.

Functional taxonomic groups are often linked with suites of traits, allowing for an extrapolation of results beyond a particular ecosystem. Herbaceous-dominated systems such as grasslands, deserts, tundra, and marshes are often dominated by distinct functional groups (e.g., C_3_ grasses), and traits associated with these functional groups may influence ecosystem-scale responses, such as shifts in net primary productivity and carbon sequestration, to interacting global change factors^10^. Coastal saltmarshes in North America are typically dominated by C_4_ grasses (e.g., *Spartina patens* (Aiton) Muhl.) or C_3_ sedges (e.g., *Schoenoplectus americanus* (Pers.) Volkart ex Schinz & R. Keller). However, an introduced lineage of the C_3_ grass *Phragmites australis* (Cav.) Trin. ex Steud. (common reed) is invading coastal and other wetlands throughout North America^45^, likely altering their response to global change^22^.

We investigated the N uptake kinetics of three functionally distinct foundation plant species in North American coastal wetlands under ambient and elevated CO_2_ conditions, and related these results to the growth of each species in response to global change factors with data from long-term *in situ* experiments. Specifically, we asked three questions:

1. Does N uptake capacity differ between *Phragmites australis*, *Schoenoplectus americanus*, and *Spartina patens*? Plants adapted to low nutrient environments typically invest considerably in belowground organs and consequently do not have a high maximum uptake capacity^46^. Given that *S. patens* invests in belowground organs to a lesser extent than *P. australis* and *S*. *americanus*, we predicted that it would have a higher maximum uptake capacity.
2. Does elevated CO_2_ affect N uptake kinetics? Given the decline in tissue N status observed in many plants grown under elevated CO_2_ despite adequate N supply, we predicted that elevated CO_2_ would negatively affect the uptake kinetics of NO_3_^−^ and NH_4_^+^ in all three species.
3. Do N uptake kinetics of our species explain observations in the field? We hypothesized that patterns in N uptake kinetics would correspond to species’ productivity responses to both CO_2_ and N in long-term field experiments.

## Results

### Kinetics of NO_3_^−^ and NH_4_^+^ uptake

**Figure 1:**
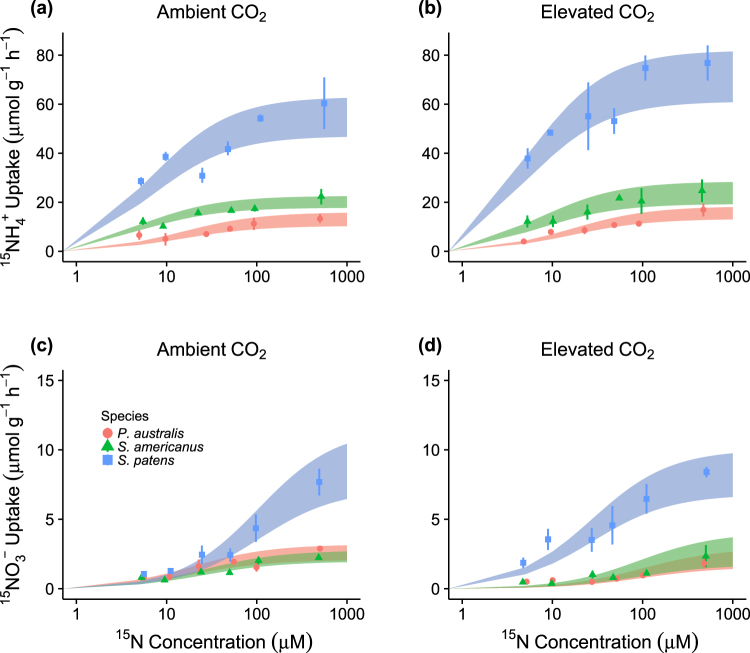
Rates of ^15^N uptake by *Spartina patens*, *Phragmites australis*, and *Schoenoplectus americanus* during assays. Points are means (±SE) for replicate plants at the six N concentrations used; horizontal jitter has been added to reduce overlap. Shaded bands show the range of Michaelis-Menten curves corresponding to the bootstrapped 95% confidence interval for V_max_.

We performed a series of ^15^N uptake assays to test the hypothesis that saltmarsh species from contrasting functional groups (C_4_ grasses, C_3_ sedges, and C_3_ grasses) would differ in their N uptake characteristics. In a semi-controlled outdoor setting, we presented clonally propagated plants with varying concentrations of either ^15^NO_3_^−^ or ^15^NH_4_^+^ and measured rates of N uptake by their root systems. All three species showed curvilinear relationships between N uptake rate (V_uptake_) and N concentration that closely adhered to Michaelis-Menten reaction kinetics (Fig. 1). Moreover, all three species exhibited substantially greater V_uptake_ for NH_4_^+^ than NO_3_^−^, with rates differing by up to a factor of 10.

**Figure 2:**
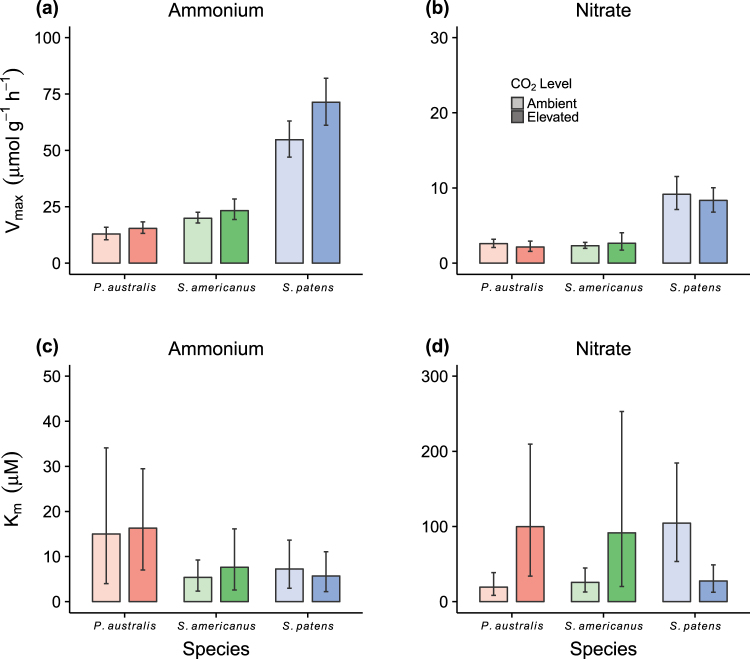
Median of bootstrapped estimates for the Michaelis-Menten parameters V_max_ and K_m_. Error bars depict the central 95% of estimates from across all bootstrapped fits (n = 999).

There were interspecific differences in V_uptake_ for both NH_4_^+^ and NO_3_^−^ (Table 1). *S. patens* was primarily responsible for these differences, as it exhibited mean uptake rates up to 3 times greater than those of *P. australis* or *S. americanus* (Fig. 1a,c) and separated from both species in pairwise comparisons (Table 2). In addition, for NH_4_^+^, *S. americanus* had 20–30% greater mean V_uptake_ across the range of N concentrations than did *P. australis* (Fig. 1a). These interspecific differences also manifested in the parameter V_max_, the maximal uptake rate, when Michaelis-Menten curves were fit to the data; in this context, V_max_ reflects a species’ capacity for N uptake under saturating N conditions. Using bootstrapped 95% confidence intervals (CIs), we again found that *S. patens* had greater V_max_ than either of the C_3_ species for both NO_3_^−^ and NH_4_^+^ (Fig. 2a,b), and that *S. americanus* had a greater V_max_ for NH_4_^+^ than did *P. australis* (Fig. 2a).

**Table 1:** Results of linear modeling analysis for nitrogen uptake (V_uptake_).

Model	Term	d.f.	F	*P*
**NH**_**4**_^**+**^ **uptake**	**Species**	**2**	**278.38**	**<0.001**
	**CO**_**2**_	**1**	**10.44**	**0.002**
	**N Conc**	**1**	**26.17**	**<0.001**
	**Species** × **CO**_**2**_	**2**	**3.97**	**0.02**
	Species × N Conc	2	1.74	0.18
	CO_2_ × N Conc	1	0.18	0.68
	Species × CO_2_ × N Conc	2	0.06	0.93
**NO**_**3**_^−^ **uptake**	**Species**	**2**	**87.78**	**<0.001**
	CO_2_	1	0.05	0.83
	**N Conc**	**1**	**111.94**	**<0.001**
	**Species × CO**_**2**_	**2**	**15.26**	**<0.001**
	**Species × N Conc**	**2**	**8.42**	**<0.001**
	CO_2_ × N Conc	1	0.31	0.58
	Species × CO_2_ × N Conc	2	0.82	0.44

**Table 2:** Mean rates of inorganic N uptake (V_uptake_; µmol g^−1^ h^−1^) across N concentrations by the three focal species. Group letters that differ within an N form denote statistical separation in pairwise comparisons of means.

**Species**	**CO**_**2**_ **Level**	**NH**_**4**_^**+**^			**NO**_**3**_^**−**^		
		**Mean**	**SE**	**Group**	**Mean**	**SE**	**Group**
*P. australis*	Ambient	9.09	2.11	a	1.60	0.24	b
	Elevated	10.29	2.04	a	0.88	0.27	a
*S. americanus*	Ambient	16.12	2.04	b	1.36	0.24	ab
	Elevated	17.76	1.98	b	1.06	0.25	ab
*S. patens*	Ambient	42.91	1.98	c	3.30	0.24	c
	Elevated	57.34	2.04	d	4.81	0.24	d

We carried out an additional set of assays under elevated CO_2_ to determine how the N uptake kinetics of our focal species could shift under future atmospheric conditions. We found that elevated CO_2_ altered patterns of N uptake, though these effects were species-specific and differed by N form and concentration (Table 1, Figs 1 and 2). For NH_4_^+^, mean V_uptake_ increased under elevated vs. ambient CO_2_, with *S. patens* showing the greatest, and statistically unequivocal, increases; the other two species exhibited non-significant trends towards increases as well (Fig. 1, Table 2). Also, *S. patens* continued to have greater V_uptake_ and V_max_ for both NH_4_^+^ and NO_3_^−^ than both C_3_ species when grown under elevated CO_2_, and the separation between *S. americanus* and *P. australis* in these metrics was maintained under elevated CO_2_ (Fig. 2a,b, Table 2). However, for NO_3_^−^, elevated CO_2_ induced a reduction in mean V_uptake_ for *P. australis*, such that it was not differentiable from *S. americanus* in either CO_2_ setting (Fig. 1c vs. d, Table 2).

For NO_3_^−^, interspecific differences in V_uptake_ depended on N concentrations and the CO_2_ level (Table 2), with CO_2_ inducing larger shifts within species at low N concentrations (Fig. 1c,d). Correspondingly, there was no evidence of CO_2_ affecting V_max_ in any species, whereas it induced notable shifts in the Michaelis-Menten parameter K_m_ (Fig. 2d). In the context of this study, K_m_ reflects a species’ affinity for an N form, with smaller values indicative of greater affinity. The shift in K_m_ under elevated CO_2_ was again greatest (and statistically unequivocal) for *S. patens*; bootstrapped CIs did not overlap. *S. patens* thus had a greater affinity for NO_3_^−^ under elevated CO_2_ (Fig. 2d). Although the corresponding 95% CIs for *P. australis* were partly overlapping and the three-way interaction was not significant for V_uptake_ (Table 1), our data were consistent with *P. australis* experiencing the opposite shift, namely a decrease in affinity (i.e., an increase in K_m_) for NO_3_^−^ under elevated CO_2_ (Fig. 2d). The data were likewise statistically equivocal for *S. americanus* (i.e., CIs were overlapping), as were CIs for all species with respect to NH_4_^+^ (Fig. 2c).

### Growth responses to global change factors

**Figure 3:**
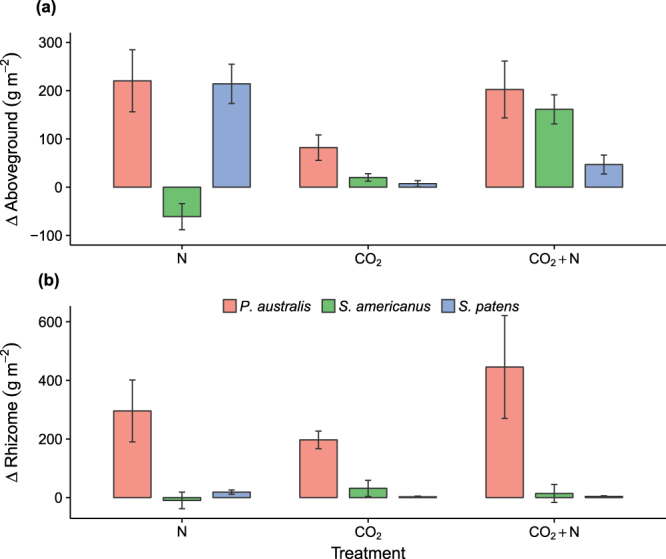
Mean (±SE) stimulation effects (i.e., difference from ambient experimental treatments) for (**a**) aboveground biomass production and (**b**) rhizome biomass production in experimental plots Global Change Research Wetland. The three treatments were *CO*_2_, elevated atmospheric CO_2_; *N*, nitrogen fertilization; and *CO*_*2*_ + *N*, both elevated CO_2_ and N fertilization. Means are calculated using the first 5 years’ worth of data from two long-term field experiments (see text).

To determine if N uptake kinetics can explain species responses to inorganic N eutrophication in the field, we compared the results of our assays with data on biomass production (aboveground and rhizome) from two long-term field experiments in which elevated CO_2_ and NH_4_^+^ were added factorially to plots in a Chesapeake Bay saltmarsh^10,22^. In the first five years of these experiments’ lifespans, N enrichment positively stimulated aboveground biomass production by *S. patens* and *P. australis* (by 214 and 220 g m^−2^, respectively; Fig. 3), with stimulation defined as the absolute difference in productivity between treatment and control conditions. In contrast, *S. americanus* responded negatively to N enrichment (−61 ± 27 g m^−2^; mean ± SE). Elevated CO_2_ positively stimulated aboveground biomass production for all three species, with the inter-annual mean change being greatest for *P. australis* (82 ± 26 g m^−2^), intermediate for *S. americanus* (20 ± 7 g m^−2^), and smallest for *S. patens* (7 ± 1 g m^−2^; Fig. 3a). Responses to the combined treatment (elevated CO_2_ + N) were likewise greatest for *P. australis* and *S. americanus*; they produced 202 and 161 g m^−2^ more than they did under the control, respectively (Fig. 3a). Belowground, *P. australis* also had the strongest growth responses to all three treatments, with the largest mean stimulation to rhizome biomass production observed under elevated CO_2_ + N (445 ± 175 g m^−2^, Fig. 3). N enrichment did not affect rhizome biomass stimulation of *S. americanus* (−10 ± 28 g m^−2^) and *S. patens* responded more strongly belowground to N enrichment (18 ± 7 g m^−2^) than to the other two treatments (Fig. 3).

## Discussion

Our results suggest that plant responses to interacting global change factors may be related to differences in N acquisition kinetics among plant functional groups. In a prior analysis of the native saltmarsh community’s response to CO_2_ and N at our site, C_4_ grasses respond strongly to N addition^10^, demonstrating that N-induced plant community shifts can alter the ecosystem’s productivity response to elevated CO_2_. Our data suggest that this shift may be attributable to a difference in the N uptake capacity of the dominant C_3_ and C_4_ species in the community (*S. americanus* and *S. patens*, respectively). A high capacity for nutrient uptake, V_max_, is considered to be an adaptation to nutrient rich conditions, whilst a low K_m_ denotes a high affinity for the substrate^7^. Here, V_max_ levels for NH_4_^+^ uptake under ambient conditions were 150% higher in *S. patens* than in *S. americanus*, indicating that it is a high-nutrient species capable of taking advantage of N enrichment (Figs 1–3). In contrast, *S. americanus* is a low nutrient specialist (evidenced by low V_max_ and low K_m_), and has a limited ability to take advantage of increased soil N (Fig. 3). Furthermore, plant species with a high V_max_ generally do not produce a high root length density and are therefore competitively inferior when nutrients in soil solution are chronically low^46^. Consistent with this pattern, fine root production was, on average, twice as high in stands of *S. americanus* than in stands of *S. patens* over the past 20 years^23^. Given this, the divergent response of these two North American wetland species to elevated N is likely attributable to differences in their N uptake kinetics, and can therefore be used in a predictive framework to project plant community shifts in response to global change.

Plasticity in N uptake physiology may explain the ability of *P. australis* to thrive in both resource-poor and resource-rich habitats. For example, our results and those of a prior study^47^ suggest that *P. australis* is adapted to a low N environment, given its low V_max_. However, intermediate V_max_ levels have been measured in *P. australis*^13,48^, as have levels an order of magnitude greater than we found^49^. As suggested by Romero *et al*.^49^, the ammonium uptake kinetics of *P. australis* seem to be plastic, such that they can be modified in response to nutrient availability or CO_2_ availability. The plastic response of N uptake to varying CO_2_ and N levels in the field study may partly explain why introduced *P. australis* can thrive under both high and low nutrient environments^50^. Our results provide evidence that *P. australis* has the kinetic parameters needed to invade low nitrogen environments, whilst our long-term field study shows that the species can thrive in resource rich environments. Furthermore, our long-term study clearly shows that *P. australis* can take advantage of both CO_2_ and N, with aboveground biomass stimulated most strongly by N addition, and belowground biomass stimulated most strongly by CO_2_ + N (Fig. 3).

Elevated CO_2_ affected saltmarsh functional groups differently. Both C_3_ species (*P. australis* and *S. americanus*) exhibited a trend for lower affinity for NO_3_^−^ under elevated CO_2_ conditions, evidenced by increases in K_m_ values. The functional group-specific effect of elevated CO_2_ on NO_3_^−^ uptake capacity corresponds to differences in NO_3_^−^ assimilation previously reported by Bloom *et al*.^32,43^ and may be attributable to the reduction in photorespiration that C_3_ plants experience under elevated CO_2_ conditions, as this decreases the reductant available to power the first step of NO_3_^−^assimilation^42^. Conversely, evidence of this repression was not observed in the C_4_ species, for which K_m_ values actually decreased under elevated CO_2_. Again, elevated CO_2_ conditions appeared to reduce NO_3_^−^ assimilation in C_3_, but not C_4_, plants.

It is now well established that the active process involved in ion uptake by plant roots at relatively low nutrient levels (10–200 µM) is provided by the high affinity transport system (HATS)^8,51,52^. This transport system is used by plants growing in natural and semi-natural ecosystems^9,53^ and is likely the one operating at the concentrations observed in our field experiment^54^. The HATS for both NO_3_^−^ and NH_4_^+^ is subject to regulation in response to changes in external N availability or in the N demand of the whole plant^55^. However, the mechanisms underlying the suppression of N uptake under elevated CO_2_ remain unclear.

We found that elevated CO_2_ enhanced the physiological capabilities of our C_4_ species, such as increasing V_uptake_ of NH_4_^+^. This suggests that some C_4_ plants may become more competitive for N with near-future global change. Furthermore, this taxa-specific nutrient uptake response to elevated CO_2_ may influence differences in growth rate, as rates of N acquisition are often positively correlated with growth rates^56–58^. Indeed, *S. patens* had the kinetic parameters of an exploitative, fast growing species^59^, and data from the long-term field experiment show that its shoot biomass response to elevated N was on par with that of the invasive species *P. australis*. These physiological enhancements induced by elevated CO_2_ may also explain the stimulation effects of CO_2_ observed in C_4_ species at a 30 year experiment at our field site^23,60^. However, rapid NH_4_^+^ uptake does not necessarily translate into rapid growth; Zerihun & BassiriRad^33^ found that the relative growth rate of *Acer negundo* was unaffected by high CO_2_ despite experiencing a two-fold increase in root NH_4_^+^ uptake capacity in response to high CO_2_.

Elevated CO_2_ appeared to repress NH_4_^+^ uptake affinity in our dominant C_3_ species; this trend may help explain long-term observations in our field experiment. The increasing K_m_ values for NH_4_^+^ under elevated CO_2_ could partly explain the reduction of tissue N levels experienced by foundational saltmarsh plants^60^. In some species, such as wheat, the reduction occurs despite the supply of high doses of nitrogen^30^ indicating that the observed reduction in tissue nitrogen with elevated CO_2_ is not due to a low nitrogen concentration in the root medium, but is related to aspects of uptake itself. Indeed, N addition did not sustain the initial positive CO_2_ stimulation of C_3_ biomass in one of our *in situ* experiments^10^. This was partially explained by competition with *S. patens*, but may also be attributable to sustained CO_2_ enrichment having a gradual decreasing effect on NH_4_^+^ uptake capacity. In addition, the combined effects of N and CO_2_ on *P. australis* shoot biomass was smaller than the effect of N alone, suggesting a potential negative effect. Another contributing factor may indeed be the reduction of transpiration-driven mass flow of N through soils due to a reduction in stomatal conductance usually experienced by plants under elevated CO_2_ conditions^29^. Alternatively, the pattern may derive from reductions in root respiration, given that greater tissue N content entails greater maintenance respiration^61^ and the fact that the energy requirements for NH_4_^+^ and NO_3_^−^ uptake and assimilation constitute a significant portion of root respiration^62^. Reductions in root respiration as a result of elevated CO_2_ exposure have been reported in the literature^63^ but no satisfactory mechanisms to explain these effects have been demonstrated^64,65^.

As is the case for NO_3_^−^ uptake, the mechanisms underlying the suppression of NH_4_^+^ uptake under elevated CO_2_ remain uncertain. What is clear is that carbon metabolites such as glutamine can suppress the expression of genes associated with the HATS for both NO_3_^−^ and NH_4_^+^^66–68^. Additionally, glucose supply to plant roots can inhibit the induction of some enzymatic proteins such as glutamate dehydrogenase and asparagine synthetase^69^, both of which are involved in N metabolism^70^. The fact that C and N metabolism are tightly linked is inescapable^71^, and it may be the case that increased carbohydrate supply to roots as a result of elevated CO_2_ exposure may act directly or indirectly on plant nitrogen pools, ultimately causing a downregulation of genes associated with N uptake.

The extent to which N uptake is influenced by edaphic factors such as oxygenation of the rhizosphere, salinity conditions, or sulfide concentration was not investigated in this study. To evaluate uptake free of these effects, assays were conducted in aerobic solutions free from Na or hydrogen sulfide_._ Salinity is known to inhibit N uptake in *Spartina alterniflora* and *P. australis* by up to 40%^13,17,48^, especially at levels above 20 ppt. Similarly, anoxic conditions and hydrogen sulfide can inhibit N uptake^18,48^. How these factors influence N uptake in the field is unknown, although *S. americanus* has the ability to oxygenate its rhizosphere and can tolerate frequent flooding whilst *S. patens* inhabits higher saltier zones^72^. Therefore each species is specifically adapted to tolerate one of these confounding factors.

The interspecific differences in N uptake kinetics identified here provide an explanation for how individual plant-level responses to global change factors (such as CO_2_ and N enrichment) translate into species dynamics at a community level. We suggest that the ecosystem-level response to interacting global change factors can be related to the root uptake kinetics of N acquisition by different plant functional groups. Our results further demonstrate that *P. australis* is capable of invading low nitrogen ecosystems, whilst our long-term field study shows that it can also thrive in resource rich environments. Consequently, physiological plasticity in the invasive species appears to facilitate its proliferation. Further study is required to determine if rising atmospheric CO_2_ levels can be expected to repress N uptake in other ecosystems and to examine the specific mechanisms involved.

## Methods

### Nitrogen uptake assays

Three wetland taxa were selected for this study: *Schoenoplectus americanus*, *Spartina patens*, and a lineage of *Phragmites australis* subsp. *australis* (haplotype M). All three are highly abundant in saltmarshes along the Atlantic Coast of North America and are representative of the plant functional groups that dominate tidal marshes, namely C_3_ sedges, C_4_ grasses, and C_3_ grasses, respectively. *P. australis* subsp. *australis* is both introduced and invasive in North America^73^, while the two natives are dominant species in two long-term experiments situated at the Smithsonian Environmental Research Center (SERC) in Maryland, USA.

The nutrient uptake experiment was conducted in a set of six chambers (1.0 × 0.7 × 1.0 m) located at Bryn Mawr College in Pennsylvania, USA (40.0297°N, 75.3139°W). During the course of the experiment, plants experienced natural temperature fluctuations, with a mean daily high of 29.8 ± 0.8 °C and a mean daily low of 19.5 ± 0.5 °C. The chambers had closed walls constructed of Lexan polycarbonate, though they were not air-tight. Blowers continuously moved air into chambers at a rate that replaced the volume of each chamber once approximately every two minutes. Three chambers were maintained at ambient CO_2_ and three at elevated CO_2_ (ambient +300 ppmv CO_2_). CO_2_ concentrations in the chambers were monitored with CM-0212 CO_2_ loggers (CO_2_ Meter, Ormond Beach, USA) and adjusted manually on a daily basis.

Plant material was collected in the spring of 2012 from SERC, maintained for one year in the Bryn Mawr College greenhouse, and propagated from rhizome fragments or emergent shoots in May 2013. Propagules were washed clean of organic matter and dead root material, and individual shoots were placed in square pots (10 cm sides) filled with clean sand to facilitate transfer to a hydroponic medium during N uptake assays. Thirteen plants per species were placed in each chamber in June 2013 (n = 234 total plants) and fertilized weekly with a 1/10^th^ strength Hoaglands solution. Within 10 weeks, individual plants achieved a root mass suitable for assays (>100 mg dw).

To investigate NH_4_^+^ and NO_3_^−^ uptake kinetics, we presented individual plants with a ^15^N-labeled substrate in hydroponic solution. The protocol for assays was adapted from Epstein *et al*.^74^ and Mozdzer *et al*.^13^. Briefly, plants were washed free of sand and placed in an N-free solution of 0.50 mM CaCl_2_ overnight to maintain root epidermal cell integrity. After equilibration, each plant was exposed to one of six different N concentrations (5, 10, 25, 50, 100, and 500 μΜ) of either ^15^NH_4_Cl or K^15^NO_3_, respectively (99% enriched; Cambridge Isotope Laboratories, Andover, USA) for 45 minutes in a well-mixed 0.50 mM CaCl_2_ solution. To ensure that drawdown would not exceed 10% of the starting concentration, the reaction volume for assays was adjusted to 2500 ml for the lowest two concentrations and 1000 ml for the remaining concentrations. The treatment assay solution was identical to the equilibration medium but contained the labeled N dose. Each exposure series for both forms of N was applied to the three species in each chamber, such that the complete set of assays was performed in triplicate (n = 216 plants). One additional plant per species from each chamber was exposed only to the equilibration medium as a control (n = 18 plants). After 45 minutes of exposure, roots were rinsed for 2 min with 1 mM KCl to remove any excess labeled substrate from root surfaces. Each plant was then separated into root, rhizome, and stem tissue and dried at 60 °C to constant weight. Dry tissue was ground using a Retsch Mixer Mill 400 (Verder Scientific, Haan, Germany). To minimize potential effects of diurnal variation in nutrient uptake, assays were conducted at approximately the same time each day (1000–1200 h) over the course of three weeks, with the three exposure series for one plant species, one N form, and one CO_2_ level (n = 18 plants) completed per day. Samples of root tissue were analyzed for ^15^N using a Europa Integra continuous flow mass spectrometer (UC Davis Stable Isotope Facility).

Uptake rates of ^15^N (V_uptake_) for individual plants were calculated from the mass of ^15^N that they assimilated (m_assim_, in µg)^13,75^:

1 $${m}_{assim}=\frac{{m}_{1}(AP{E}_{samp}-AP{E}_{ctrl})}{AP{E}_{treat}}$$

2 $${V}_{uptake}=\frac{({m}_{assim}/M{W}_{treat})}{\,({m}_{2}\,{t}_{exp})}$$

where *m*_1_ is the mass of N in the sample (in μg), APE_samp_ is the atom % excess ^15^N of the root sample exposed to a labeled substrate, APE_ctrl_ is the atom % excess ^15^N in the control root sample, APE_treat_ is the atom % excess of the labeled ^15^N treatment, MW is the molecular weight of the N isotope, *m*_2_ is the dry root mass of the sample (in grams), and *t*_*exp*_ is the duration of the exposure to labeled substrate (in minutes). Several uptake rates were anomalously high, especially at low N concentrations (5–25 μM). This was probably due to carryover during mass spectrometry, so V_uptake_ values that were greater than those at both of the next two higher N concentrations within a series were omitted (n = 19).

The ^15^N uptake rates from each exposure series (n = 14–18 plants) were then fit to the Michaelis-Menten equation in order to derive values of maximal uptake rate (V_max_) and the substrate concentration at which the rate is 50% of V_max_ (K_m_):

3 $${V}_{uptake}=\frac{{V}_{max}[c]}{{K}_{m}+[c]}$$

where [c] is the concentration of NH_4_^+^ or NO_3_^−^. V_max_ (in µmol ^15^N g^−1^ h^−1^) provides a measure of uptake capacity under saturating N conditions, while K_m_ (in µM of NH_4_^+^ or NO_3_^−^) provides an estimate of the species’ affinity for NH_4_^+^ or NO_3_^−^; smaller values correspond to greater affinity. Curve fitting was carried out in R using a self-starting non-linear regression function (*SSmicmen* from the *nlstools* library). To determine if there were differences among species and/or CO_2_ levels, we used bootstrapping to compute 95% confidence intervals for all parameter estimates (via *nlsBoot*, also from *nlstools*; n = 999 iterations). Estimates were considered different if there was no overlap between pairs of bootstrapped 95% confidence intervals^76^.

Linear models were used to determine how experimental factors (CO_2_ level, plant species, and N concentration) affected N uptake rates (V_uptake_), with separate models fit to data for NO_3_^−^ and NH_4_^+^. Both models had the same form, with species and CO_2_ level included as categorical variables but N concentration included as a continuous variable. Terms for all possible two and three way interactions were also included. V_uptake_ values were square root transformed to ensure residual normality. Tukey-adjusted pairwise comparisons were subsequently made among all species-CO_2_ level combinations; the family-wise error rate was held at 0.05. All statistical analyses were conducted in R version 3.2.3.

### Long-term field experiments

We compared results from our *ex-situ* kinetic experiment with *in situ* data from two long-term experiments situated in a brackish tidal marsh within SERC’s Global Change Research Wetland (Kirkpatrick Marsh; 38.8742° N, 76.5474°W) in Edgewater, Maryland, USA. The first experiment was established in 2006 and examines the effect of elevated CO_2_ and mineral N addition on the dominant native saltmarsh species *S. americanus* and *S. patens*. The second study was established in 2011 and examines the effect of identical global change manipulations on the introduced lineage of *P. australis* (haplotype M) and its encroachment into the native marsh community. For details of chamber setup and experimental design see Langley and Megonigal^10^ and Caplan *et al*.^22^. Briefly, half of the plots in each experiment are fertilized with NH_4_Cl at a rate of 25 g N m^−2^ yr^−1^ and half of the plots at each N level are fumigated with sufficient CO_2_ to raise the atmospheric concentration within open-top chambers by approximately 300 ppm throughout the growing season (May through November).

Aboveground biomass is estimated in both experiments in late July or early August of each year. For *S. americanus* and *P. australis*, this entails combining stem density counts with measurements of stem height and width that are converted to dry mass using species-specific allometric relationships^77^. For *S. patens*, biomass is measured directly by clipping samples within each chamber. Rhizome productivity is estimated from annual samples collected each year using ingrowth cores, with three placed in each plot for the native marsh study and six placed in each plot for the *P. australis* study.

Biomass data from the two long-term field experiments were used to quantify productivity responses to global change factors. Specifically, we calculated stimulation effects (i.e., differences from ambient) for the elevated CO_2_ treatment, the N enrichment treatment, and the combination treatment for both aboveground biomass and rhizome biomass. For the native marsh study, we used biomass data from *S. americanus* and *S. patens* spanning the first five years that data were available (2006–2010 for aboveground biomass and 2007–2011 for rhizome biomass). Biomass data for *P. australis* came from the second study, but likewise spanning the first five years of its lifespan (2011–2015).

### Data availability

The datasets used in this study are available from the corresponding author on reasonable request.
